# Simple Climate Models That Can Be Used in Primary, Secondary, and Tertiary Education

**DOI:** 10.1021/acs.jchemed.4c00541

**Published:** 2024-08-30

**Authors:** Timothy G. Harrison, Michael T. Davies-Coleman, Alison C. Rivett, M. Anwar H. Khan, Joyce D. Sewry, Magdalena Wajrak, Nicholas M. Barker, Jonny Furze, Sophie D. Franklin, Linda Sellou, Naomi K. R. Shallcross, Dudley E. Shallcross

**Affiliations:** 1 School of Chemistry, Cantock’s Close, University of Bristol, Bristol BS8 1TS, U.K.; 2 Department of Chemistry, University of the Western Cape, Robert Sobukwe Road, Bellville, 7535, South Africa; 3 Department of Chemistry, Rhodes University, Makhanda, 6139, South Africa; 4 School of Science, 270 Joondalup Drive, Edith Cowan University, Perth, WA 6027, Australia; 5 Social Inclusion Group, University of Warwick, Coventry, CV4 7AL, U.K.; 6 Primary Science Teaching Trust. 12 Whiteladies Road, Bristol, BS8 1PD, U.K.; 7 Department of Chemistry, National University of Singapore, Singapore 119077; 8 Becket Primary School, Tavistock Rd, Worle, Weston-super-Mare BS22 6DH, U.K.

**Keywords:** Audience, General Public, Elementary, Middle School Science, High School, Introductory Chemistry, First-Year Undergraduate, General, Second-Year Undergraduate, Upper-Division Undergraduate, Graduate Education, Research: Continuing Education, Domain, Demonstrations, Environmental Chemistry, Interdisciplinary, Pedagogy, Multimedia-based learning

## Abstract

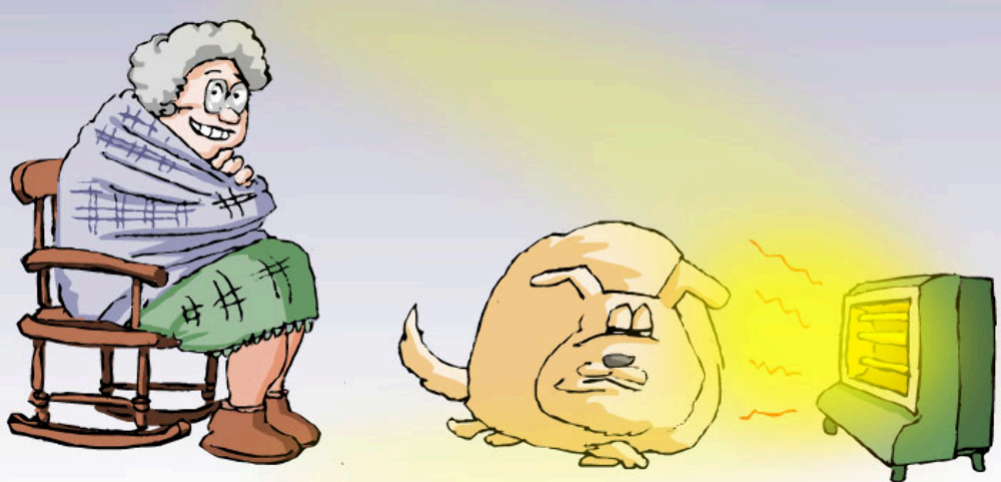

Climate change is of great concern to all age groups but in particular to children. “Simple” climate models have been in place for a long time and can be used effectively with post-16 students. For younger children, modifications are required, and we describe in this paper the development and use of two such models. The first (the Granny Model) is a pictorial version of the model that has been used extensively with primary and early secondary school aged children (14 and younger). The second is an online version of the simple climate model that can be used without recourse to the underpinning mathematics and science but allows children to experiment with changing variables and how these changes affect the average surface temperature of the Earth.

## Introduction

It is generally assumed that models of the Earth’s climate are complicated and cannot be addressed at secondary school or college level (e.g., 11–18 years old in the UK) let alone at primary or elementary school level (e.g., 4–11 years old in the UK). However, some well-known “simple” models of the Earth’s atmosphere and climate system^1^ can be used and modified to allow school students and indeed undergraduates and postgraduates to be able to investigate the Earth’s climate. In this way, these students can carry out their own investigations and explore the Earth’s climate system. Climate change is a very pressing issue, and as part of the Bristol ChemLabS Centre for Excellence Outreach program,^2−4^ we have developed a range of climate related resources and used these in schools. In this article we describe two models we have developed, the first one is a pictorial model, we call the “Granny” model that can be used for any age of learners (we have used it with primary school children in the UK, aged 7 and older), and the other is a dynamic online model that students can use to determine changes in surface temperature based on changes to the incoming solar energy, the amount of clouds in the sky and the amount of surface ice on the Earth, and the amount of Greenhouse gases and the amount of ozone in the stratosphere. Other online models of a similar structure exist, but we have worked with primary and secondary school teachers to produce an online model that is as simple as possible and can potentially be used in schools (it has been used by secondary schools in the UK) and by the general public. There is no doubt that climate change is one of the most pressing concerns we face currently^5^ and school children and the general public welcome the opportunity to be able to explore and understand the system better.^6^ We will describe the two models, and the underpinning climate science is presented in the Supporting Information.

We note that online models have been developed to aid the understanding of climate change but tend to focus on teachers and upper secondary and tertiary students.^7^ Models using excel have also been used to investigate past climate data and variations in greenhouse gases (CO_2_, CH_4_, and N_2_O).^8^ Several projects have developed experiments that are contextual for post-16 and tertiary curricula that support the understanding of elements concerned with climate change,^9,10^ while others include videos.^11^ Mapping of general chemistry concepts to those related to underpinning the Chemistry associated with the Earth’s climate has been carried out quite recently and “rich contexts” generated for these topics through the lens of the Earth’s climate.^12^ For primary (elementary) school children and their teachers, resources tend to focus on exemplar experiments that convey aspects of climate change, e.g., the Greenhouse Effect.^13^

## Granny Model

**Figure 1:**
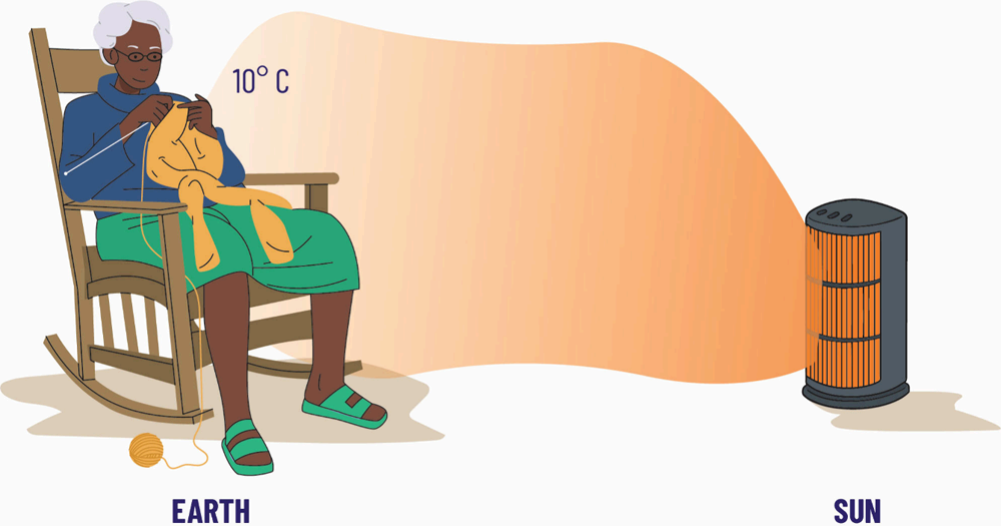
First Granny model, with just Granny and a heater, in an energy flux calculation shows that the average surface temperature of the Earth is around 10 °C.

The pictorial Granny Model has been used with primary, secondary, and tertiary students in the UK and with secondary and tertiary students in South Africa, Australia, and Singapore for the past 10–15 years. There is evidence that a Granny has a rather unique and positive place in young children’s thinking and so we have used Granny as the character that takes the children on a journey through the Earth’s climate system.^14,15^ The set of images used over the years is essentially the same in each case, but we have modified Granny so that she is contextual for the group being engaged. If we imagine that Granny is in a room and there is a heater (Figure 1), Granny knows that if she sits too close to the heater, she will be too hot, too far away she will be too cold, and so Granny places herself a sensible distance from the heater, so that she is neither too hot, nor too cold (sometimes called the Goldilocks’ zone). This is the first interpretation of the Granny model of the Earth’s climate, where Granny represents the Earth, and the heater is the Sun. The Earth is not too far away or too close to the Sun, so liquid water can exist on the surface. It turns out that (see Supporting Information) that this simple model returns an average surface temperature of the Earth of about 10 °C, which is a little cool compared with reality. However, this demonstrates that the first and most important component of the Earth’s climate is the distance from the Sun which then determines the energy per second per unit area that the Earth receives.

**Figure 2:**
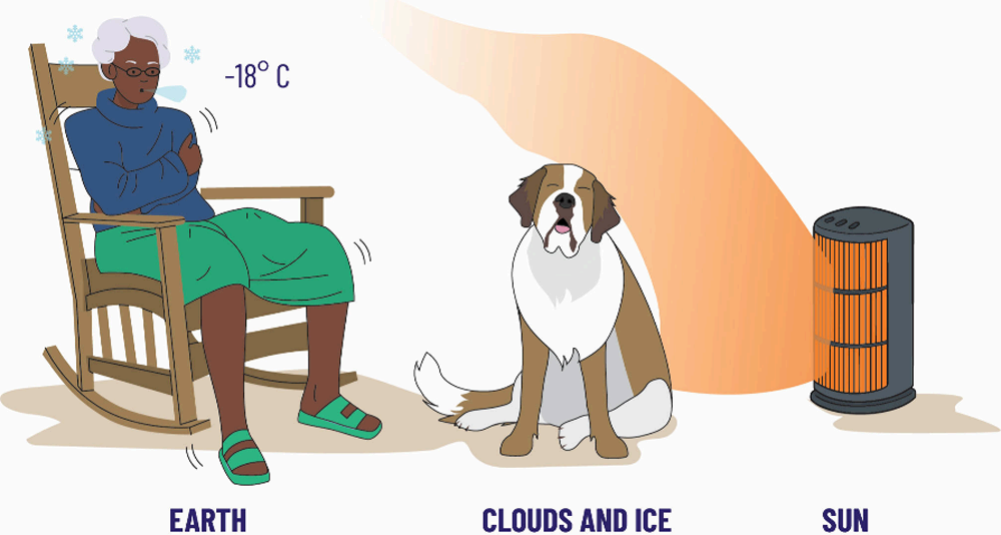
Second Granny model, where an animal sitting in front of the fire represents the effect of clouds and ice. Adding in the effect of clouds and ice reduces the average surface temperature of the Earth to −18 °C.

**Figure 3:**
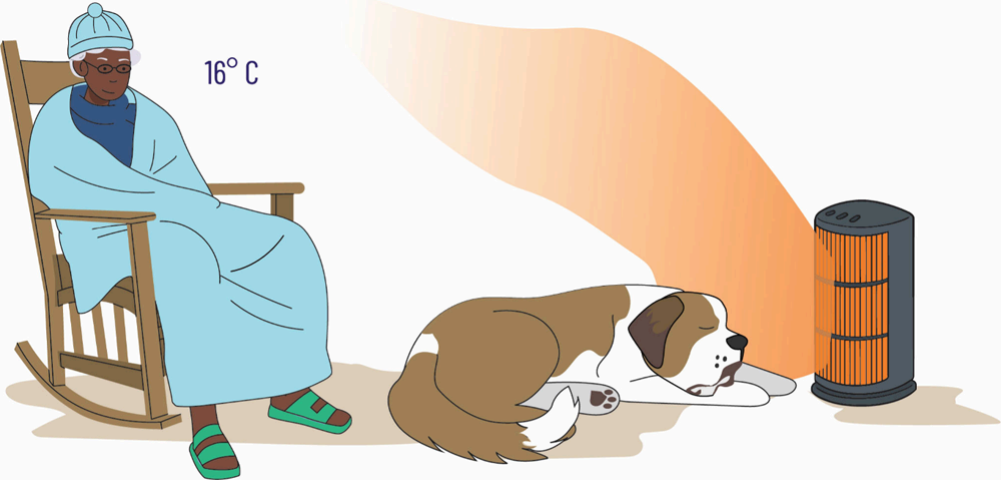
Third Granny Model, now Granny has a blanket (depicting the role of Greenhouse gases in warming the surface of the Earth). With clouds and ice and greenhouse gases, the average surface temperature of the Earth is approximately 16 °C.

However, there are clouds in the sky and ice at the Earth’s surface, and these act-like mirrors to the incoming solar radiation and so some of the energy from the Sun is reflected back to space and the energy received by the Earth (Granny) is a lot less. We depict this in the second Granny model by imagining that Granny’s pet dog has come and sat down next to the heater and is preventing some of the heat from reaching Granny, (Figure 2). We debated the underlying Physics and Chemistry of this second model. Ideally, we want to have a silvered dog, i.e., one that has a surface that reflects most of the heat it receives back toward the heater, but we have found that for young learners (KS 1–3 in the UK, i.e. up to about 14 years old) this simplified diagram makes more sense than a silver dog, which distracts from the development of the model. When we present this simple Granny model to older students, who will probably know more about energy absorption and reflection, we then follow up with the more involved mathematical model and explain the modification that would be needed. Students and teachers have informed us that the Granny model is a very good way to present the overall model before going into the mathematics. For undergraduates, we often ask them to critique the Granny model, and their replies confirm that the benefits of having a physical picture of the Earth system is far more useful, even with a physical flaw. The reflectivity of the Earth is known as its albedo and is around 30% or 0.3. That is to say, around 30% of the incoming solar energy is reflected back to space, and the average surface temperature of the Earth is now −18 °C. Granny is now very cold, and the Earth should be covered in ice; since this is not the case, as we pose to the children, what can Granny and the Earth do to warm back up again? We will have several suggestions such as move closer to the heater. We note that the Earth’s distance from the Sun is not fixed and does vary as it transitions from a roughly elliptical to a more circular orbit, so that the Earth is on average closer to the Sun (interglacial periods such as now) and further away, which leads to glacial periods where the Earth’s surface has extensive regions of surface ice (the last one (ice age) ending about 10,000 years ago). Another common question is, can we remove the dog? Since this represents water in the Earth system, we note that removing water would be a bad thing for life on Earth. Eventually, sometimes with some prompting, the children will suggest that Granny gets a blanket, and this leads to the third Granny model depicted in Figure 3. For the younger audience (typically 14 years and younger), we simply state that Greenhouse gases such as CO_2_ in the atmosphere act like a blanket. For older audiences and depending on their background knowledge, we will discuss the infrared energy spectrum emitted by the Earth and why certain gases in the Earth’s atmosphere are important Greenhouse gases. These Greenhouse gases can absorb infrared energy in the region where the Earth emits and undergo vibrations and rerelease that energy in all directions, with some of that energy being directed back to the surface, leading to the warming effect. We note that without Greenhouse gases the Earth would be much colder than it is, and for many species living on Earth, it would become uninhabitable. Therefore, Greenhouse gases are not bad; they are essential for a habitable Earth, but if the levels of Greenhouse gases become too high then the average surface temperature of the Earth will increase, and this may become a problem as we are beginning to witness now through extreme weather events. Therefore, the Granny model, in its three parts, is a very effective way to explain how the Earth’s climate system works to young learners but also to all learners unfamiliar with the topic.

## Online Simple Climate Model

The reader is directed to the Supporting Information where we provide the background mathematics that underpins the online climate model (https://bit.ly/PSTTCC1 and https://bit.ly/PSTTCC2), and we remind readers that we did not invent this model, it has been used for many years in the atmospheric science community.^1^ There are four essential components to the model, the energy from the Sun (Joules per second per m^2^), denoted by the symbol F_S_ arriving at the top of the Earth’s atmosphere. Second is the Earth’s albedo (its reflectivity), denoted by the symbol A, that can vary between 0 (not incoming solar radiation is reflected) and 1 (all incoming solar radiation is reflected). Third is τ_UV_VIS,_ this is the transmittance of incoming UV and Visible solar radiation through the Earth’s atmosphere to the surface. τ_UV_VIS_ varies between 1 (all incoming solar radiation passes through the atmosphere to the surface, and 0, where all incoming solar radiation is absorbed by the atmosphere. O_2_ and O_3_ absorb harmful UV radiation between 200 and 300 nm approximately in the stratosphere (around 10–50 km in altitude)^16^ and so a typical value is 0.8. The final component is τ_IR,_ this is the transmittance of outgoing infrared radiation emitted by the Earth’s surface. Τ_IR_ varies between 1 (all outgoing infrared radiation passes through the atmosphere to space and is not absorbed, and 0, where all outgoing infrared radiation is absorbed by the atmosphere (by Greenhouse gases). The model leads to an expression for the average surface temperature of the Earth, *T*_E_, that is a function of these four variables, i.e.
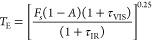
1

This expression can be incorporated into a spreadsheet very easily, and students can vary the parameters to see the effect on *T*_E_. We have done just this with post-16 students and undergraduates, but you would need to explain all the parameters (see the Supporting Information), so this activity is best as a workshop where a few hours can be dedicated. Some interesting basic results emerge and are summarized in Table 1. In experiment 1, we imagine that there are clouds and ice (*A* = 0.3) but the atmosphere does not affect either incoming solar radiation or outgoing infrared radiation from the Earth (both τ_VIS_ and τ_IR_ are 1), this then yields a very cold average surface temperature of around −18 °C and corresponds to Granny model 2. In experiment 2, we now remove clouds and ice, the average surface temperature increases to 5 °C, a rise of around 23–24 °C (this corresponds to Granny model 1) and demonstrates the strong cooling effect of clouds and ice on the Earth’s climate. In experiment 3, we imagine that we now fill the Earth’s atmosphere with Greenhouse gases, so that no infrared radiation from the Earth can escape to space. This leads to an average surface temperature of around 57 °C, which is terrifying (in terms of Granny, this is model 3 but with Granny having many blankets on, this Granny model is not used). If in experiment 4, we reintroduce clouds and ice (*A* = 0.3), and the average surface temperature becomes around 29 °C.

**Table 1:** Results from the Expression for the Average Surface Temperature of the Earth, *T*_E_, Shown in equation 1

Experiment	1	2	3	4
FS (W m^–2^)	342.5	342.5	342.5	342.5
*A*	0.3	0.0	0.0	0.3
τ_VIS_	1.0	1.0	1.0	1.0
τ_IR_	1.0	1.0	0.0	0.0
*T*_E_ (K)	254	278	330	302
*T*_E_ (°C)	–19	5	57	29

However, by creating a simple slider bar online version of the model, students and their teachers can investigate outcomes without having to understand the underpinning mathematical model. We have used a similar version at the beginning of the Outreach program, and this worked well and was used by many schools, but because of software updates it was decommissioned. This new version has been road-tested by and codeveloped with school teachers (both primary and secondary). There are two versions, the first assumes a fixed albedo of 0.3, to simplify the online model, essentially *T*_E_ now becomes eq 2
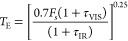
2

In the second online model, the full eq 1 is used.

## Example Challenges (for Post-16 Students)

Example questions that could be asked, that would require the students to use this model (online or spreadsheet), are

1. Which variable has the greatest effect on average global temperature? This is an open-ended question, as one would first assume that the solar constant is the most important. However, if *A* approaches 1, that is, the Earth’s surface becomes very reflective, covered in ice like the snowball Earth then the average surface temperature plummets.
2. What would happen to the average surface temperature if the ice caps melted? This question is asking the student to reduce *A* by approximately 6% to 24% or 0.24 in the calculation. It is quite surprising how much the temperature rises with modest reductions to the Earth’s albedo. Of course, more challenging questions could be asked. For example.
3. The sand in the Sahara Desert can be made into a glass mirror. If a perfectly reflecting mirror is put at the Earth’s surface in the Sahara Desert, it could reflect incoming solar radiation and cool the planet. What would the new average surface temperature be now?
  How big would the mirror have to be to cool the planet by 1 °C?
  What fraction of the Sahara Desert would that be?
  Students would need to estimate the size of the Sahara Desert relative to the surface area of the Earth and estimate what change to A would then occur.

Matthew Elrod has written an article for *J. Chem. Educ.*, on the Earth’s infrared spectrum and how Greenhouse gases interact with it. It is possible to take Elrod’s model^17^ and use that to derive τ_IR_ as a function of individual Greenhouse Gases and their atmospheric concentration. We have done this with final year undergraduates in workshops as this emphasizes why certain gases are strong Greenhouse gases (e.g., CFCs, HCFCs, and HFCs)^1^ and revises infrared spectroscopy, Beer–Lambert Law, and thermodynamics.

## Model Feedback and Top Tips

Engaging younger children with the first Granny model image is essential, i.e., orientating the children, so that they appreciate that there is a heater in a room and asking them what would happen if the Granny moved closer to the heater or further away. Once the children are thinking about the Granny getting hotter or colder, then it is possible to proceed to the second image and ask them whether Granny would warm up or cool down? It can take a while for them to decide, but they do reach a consensus that Granny will cool down. The last image is much easier to discuss, and the children will know that the blankets will warm Granny up. The problem that always exists with analogies is translating the model from Granny and a heater to Earth and the Sun. However, young children can be reminded that when clouds are present it generally leads to cooler temperatures, and so starting from a clear sky day it will cool when clouds are added. For secondary school children (11–16) in the U.K., they are building up their knowledge about heat transfer, and so it is much easier to progress through the images quite quickly. In addition, these children will have some knowledge of the solar system, and so the analogy works well. It is very important to introduce the Granny “pictorial” model first and then introduce the online model to these students. If they work in small groups and are set some tasks, most simply to work through the four experiments summarized in Table 1, then feedback suggests that these students can appreciate the key elements of the Earth system. For the general public (during science festivals for example or public events), they understand the concepts expressed in the Granny model (through feedback).

## Summary and Conclusion

Simple climate models exist that can be adapted for use with post-16 students and allow a wide range of investigations that illustrate the effect of changing key variables have on the average surface temperature of the Earth. The use of such models would help alleviate the confusion noted by the students, disentangling climate change, the greenhouse effect and stratospheric ozone.^18^ In addition, research suggests that Preservice Science Teacher training in climate change knowledge can have very positive impacts on teacher self-efficacy and hope.^19^ For younger learners, we have developed a pictorial version using a Granny as the focus of the analysis. This Granny model is also a very good way to set the scene for all audiences before describing the simple climate model. An online version of the simple climate model has been constructed that can be used in schools, and we welcome feedback on its use. Encouraging and supporting students to consider and explore climate mitigation strategies is very important and the development and implementation of models facilitates this.^20^

## Supporting Information Available

Description of the derivation of the eq 1 used to determine the average surface temperature of the Earth (*T*_E_)_._ (PDF, DOCX)
