# Supplementary material for: Simple Climate Models That Can Be Used in Primary, Secondary, and Tertiary Education
Source: J Chem Educ. 2024 Aug 30;101(10):4528–32. doi: 10.1021/acs.jchemed.4c00541 (PMC11465639; doi:10.1021/acs.jchemed.4c00541)
Supplement: Supplementary file 2 — ed4c00541_si_002.docx [file ed4c00541_si_002.docx]

Supporting information for the paper ‘Simple climate models that can be used in primary, secondary and tertiary education’

Timothy G. Harrison,^1✝^, Michael T. Davies-Coleman,^2^ Alison C. Rivett,^1^ M. Anwar H. Khan,^1^ Joyce D. Sewry,^3^ Magda Wajrak,^4^ Nicholas M. Barker,^5^ Jonny Furze,^1^ Sophie D. Franklin,^6^ Linda Sellou,^7^ Naomi K. R. Shallcross^8^ & Dudley E. Shallcross^1,2*^

1. School of Chemistry, Cantock’s Close, University of Bristol, BS8 1TS, UK.
2. Department of Chemistry, University of the Western Cape, Robert Sobukwe Road, Bellville, 7535, South Africa.
3. Department of Chemistry, Rhodes University, Makhanda, 6139, South Africa.
4. School of Science, 270 Joondalup Drive, Edith Cowan University, Perth, WA 6027, Australia
5. Social Inclusion Group, University of Warwick, Coventry, CV4 7AL, U.K.
6. Primary Science Teaching Trust. 12 Whiteladies Road, Bristol, BS8 1PD, U.K.
7. Department of Chemistry, National University of Singapore, Singapore.
8. Becket Primary School, Tavistock Rd, Worle, Weston-super-Mare BS22 6DH, UK

Corresponding Author

*E-mail: [d.e.shallcross@bris.ac.uk](mailto:d.e.shallcross@bris.ac.uk)

The Supporting information document contains a derivation of the expression for T_E_ shown in equation (1) in the main text.


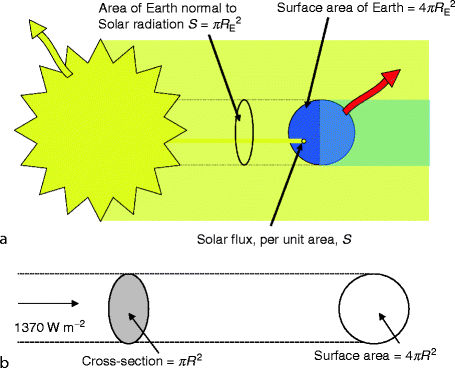


Figure S1. The balanced flux model.

Figure S1 illustrates the energy coming to the Earth from the Sun and the energy being emitted from the Earth.

Energy in 1370 Wm^-2^ x $\pi R_{E}^{2}$ S1

Energy out $\sigma T_{E}^{4} \times4\pi R_{E}^{2}$ S2

1370 Wm^-2^ is the solar constant for the distance that the Earth is on average from the Sun. This energy is distributed over a circle at the top of the atmosphere whose radius is the radius of the Earth, R_E_. So, the total energy received per second is the product of these two terms (S1)

The energy out is emitted from the whole surface of the Earth ( $4\pi R_{E}^{2}$) and the energy flux is given by the Stefan-Boltzmann equation for black body radiation ($\sigma T_{E}^{4}$), where $\sigma$, is the Stefan Boltzmann constant. At equilibrium the two fluxes, in and out, are equal (S3).

$1370 \times\pi R_{E}^{2} = \sigma T_{E}^{4} \times4\pi R_{E}^{2}$ S3

Rearranging S3 leads to the first expression for T_E_ (S4 and S5), i.e. Granny model 1.

$T_{E}^{4}=\frac{1370}{4 \times5.67 \times{10}^{-8}}$ S4

T_E_ = ($\frac{1370}{4 \times5.67 \times{10}^{-8}}$)^0.25^ S5

If we assume that only 70% (A= 0.3) of the solar constant warms the Earth’s surface, then equation S5 is modified to S6 (Granny model 2).

T_E_ = ($\frac{1370x0.7}{4 \times5.67 \times{10}^{-8}}$)^0.25^ S6

In order to take into account Greenhouse gases (Granny model 3) we need to move to a so called ‘one layer’ atmospheric model shown in figure S2.


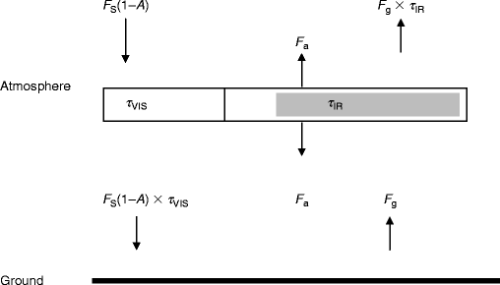


Figure S2. A schematic of the one layer atmospheric model which is described in the text.

Parameters in the one layer atmospheric model

F_s_ = solar constant / 4 ~ 342.5 Wm^-2^ (the factor of 4 arises from the ratio of the area of a disk radius R_E_ and the surface area of a sphere radius R_E_)

A – Albedo which ranges from 0-1

τ_UV_VIS_ - Transmittance of UV/Visible light from the Sun through the Earth’s atmosphere and varies from 0 – 1.

Τ_IR_  - Transmittance of infra red light from the Earth through the Earth’s atmosphere and out to space and varies from 0 – 1.

F_g_ = $\sigma T_{E}^{4}$ and is the total infra red flux from the Earth’s surface and is approximated by the Stefan Boltzmann Law.

F_a_ is the flux from the one layer atmosphere, assuming that it absorbs energy and can radiate it both upwards to space and down to the ground in equal amounts.

At the surface of the Earth, there are three fluxes, two down to the surface and one from the surface, if we assume that they are balanced we arrive at S7.

$F_{s}\left( 1-A \right)\tau VIS+F_{a}=F_{g}$ (S7)

Similarly, equating fluxes in and out at the top of the atmosphere leads to S8.

$F_{g}\tau IR+F_{a}=F_{s}\left( 1-A \right)$ (S8)

Making F_a_ the subject of both (S7) and (S8) and combining leads to S9.

$F_{g}=\frac{F_{s}\left( 1-A \right)\left( 1+\tau VIS \right)}{\left( 1+\tau IR \right)}$ (S9)

Finally, noting the Stefan-Boltzmann law once again, *F* _g_ can be expressed as

$F_{g}=\sigma T_{E}^{4}=\frac{F_{s}\left( 1-A \right)\left( 1+\tau VIS \right)}{\left( 1+\tau IR \right)}$ (S10)

Rearranging (S10) to make *T* _E_ the subject leads to S11 or equation (1) in the main text.

$T_{E}=\left[ \frac{F_{s}\left( 1-A \right)\left( 1+\tau VIS \right)}{\left( 1+\tau IR \right)} \right]^{0.25}$ (S11)
